# *Burkholderia contaminans* Colonization from Contaminated Liquid Docusate (Colace) in a Immunocompetent Adult with Legionnaire’s Disease: Infection Control Implications and the Potential Role of *Candida pellucosa*

**DOI:** 10.3390/jcm5120110

**Published:** 2016-11-30

**Authors:** Burke A. Cunha, John Gian, Bertamaria Dieguez, Elsa Santos-Cruz, Daniela Matassa, Steve Gerson, Pat Daniels, Carlos Rosales, Rodger P. Silletti

**Affiliations:** 1 Infectious Disease Division, Winthrop-University Hospital, Mineola, New York, NY 11501, USA; jgian@winthrop.org (J.G.); bDieguez@winthrop.org (B.D); 2 Infection Control Department, Winthrop-University Hospital, Mineola, New York, NY 11501, USA; ESantos-Cruz@winthrop.org (E.S.-C.); RPSilletti@winthrop.org (D.M.); 3 Pharmacy Department, Winthrop-University Hospital, Mineola, New York, NY 11501, USA; sgerson@winthrop.org (S.G.); pdaniels@winthrop.org (P.D.); 4 Medical Microbiology Laboratory, Winthrop-University Hospital, 222 Station Plaza North, Mineola, New York, NY 11501, USA; crosales@winthrop.org (C.R.); rsilletti@winthrop.org (R.P.S.); 5 State University of New York, School of Medicine, Stony Brook, New York, NY 11501, USA

**Keywords:** *Candidapullucosa*, *Burkholderia cepacia* outbreaks, *Burkholderia cepacia*, complex (BCC) colonization, *B. contaminans* outbreaks, doxycycline, levofloxacin, TMP-SMX, contaminated medications, antibiotic resistance, Legionnaire’s disease, occidiofungin *Hansenula anomala*, colonization of respiratory secretions, Gram negative bacilli (GNB), multidrug resistant (MDR)

## Abstract

**Objective:** *B. contaminans* was cultured from respiratory secretions and liquid docusate (Colace) in a Neurosurgical Intensive Care Unit (NICU) patient with community-acquired Legionnaire’s disease but not from another bottle given to the patient. Unexpectedly, *C. pelliculosa* was cultured from two bottles, but not the *B. contaminans* bottle or respiratory secretions. **Methods:** *B. cepacia*, later identified as *B. contaminans*, was cultured from a bottle of liquid docusate (Colace) dispensed to a non-cystic fibrosis patient. His respiratory secretions were colonized with *B. contaminans*. **Results:** Eradication of *B. contaminans* colonization in the patient’s respiratory secretions was attempted. With levofloxacin, *B. contaminans* developed multidrug resistance (MDR). Subsequent TMP-SMX therapy did not result in further MDR. Nine other ICU patients were given docusate from the same lot, but there were no other *B. contaminans* isolates. **Conclusion:** *B. contaminans* colonization of respiratory secretion may be difficult to eliminate. The significance of *C. pelliculosa* cultured from liquid docusate (Colace) remains to be elucidated. In this case, it appeared that *B. contaminans* may have inhibited the growth of *C. pelliculosa* in the same bottle. Others should be alerted to the possibility that *C. pelliculosa* may be present in *B. contaminans*–contaminated lots of liquid docusate (Colace).

Nosocomial *B. cepacia* or *B. cepacia* complex (BCC) colonization or infection may be acquired from a variety of contaminated aqueous sources e.g., disinfectants, mouthwash, nebulizers, intravenous solutions/medications, moisturizing cream and bacteremias, pneumonias, and urinary tract infections have rarely been reported [1,2,3,4]. Rarely, sporadic cases of *B. cepacia* endocarditis, septic arthritis, and sepsis “*B. cepacia s*yndrome” have been reported due to contaminated medical devices or liquid medications [5,6,7,8,9,10,11].

In June 2016, an ongoing multistate *B. cepacia* outbreak was reported by the Centers for Disease Control (CDC) and, as of 18 July 2016, included 49 confirmed cases from five states. The multistate *B. cepacia* outbreaks were found to be associated with contaminated liquid docusate (Colace). Cases have occurred in patients without cystic fibrosis involving critically ill patients on ventilators primarily in intensive care units (ICUs).

We report a case of *B. contaminans* colonization of respiratory secretions after receiving contaminated liquid docusate (Colace) in a critically ill patient with Legionnaire’s disease.

A 28-year-old male sustained a motor vehicle accident with severe head trauma, was admitted to the neurosurgical intensive care unit (NICU) and intubated. On hospital day (HD) #3 a chest film showed a new right lower lobe infiltrate. In addition to his chest film infiltrates, extra-pulmonary findings suggested a non-zoonotic atypical community-acquired pneumonia (CAP), most likely Legionnaire’s disease. Accordingly, he was treated empirically with doxycycline for presumed Legionnaire’s disease. The diagnosis of Legionnaires disease was confirmed by serial elevated *Legionella* sp. titers that continued to increase over two weeks, and he was switched to levofloxacin to complete therapy. On HD #7 and HD #8 his respiratory secretions were Gram-stained and cultured. The Gram stain showed some white blood cells (WBCs) and cultures were reported positive for aerobic Gram-negative bacilli (GNB). Two days later, the GNB isolated was identified as *B. cepacia*. The unusual isolate was reported to Infection Control (IC) who informed the hospital epidemiologist of the CDC alert regarding the ongoing multistate outbreak of *B. cepacia* colonization/infection in ventilated and critically ill patients.

Immediately, the hospital epidemiologist advised the pharmacy department not to dispense any further liquid docusate (Colace) to any critically ill, ICU, organ transplant, hemodialysis, or oncology patients. The index case was dispensed liquid docusate (Colace) from two bottles (bottles #1 and #2: opened/used). A third bottle (bottle #3: unopened/unused) was intended for but not given to the patient. The pharmacy department recalled the three bottles related to the index case which were transported to the microbiology laboratory for culture. The index case isolate (*B. cepacia*) was sent to the New York State Department of Health (NYS DOH) for speciation (later identified as *B. contaminans* by rec A gene sequencing at the NYS DOH Wadsworth Laboratory in Albany, New York). All unused liquid docusate (Colace) lots were returned to the manufacturer by the pharmacy department.

The index case had serial Gram stains and cultures of his respiratory secretions to determine the duration of *B. contaminans* carriage in his respiratory secretions while completing therapy of Legionnaire’s disease with levofloxacin. Blood, stool, and urine cultures were also obtained and were later reported as negative for *B*. *cepacia* (*B.* *contaminans*). The pharmacy department investigation revealed that at the time of the *B. cepacia* (*B. contaminans*) index case, nine other ICU patients had also received liquid docusate (Colace), but *B.* *cepacia* (*B. contaminans*) was not isolated from any of these or any other ICU patients. The liquid docusate (Colace) bottles used for these nine patients were discarded, and were not available for culture.

Serial chest films showed complete resolution of the patient’s community-acquired Legionnaire’s disease, as well as normalization of the non-specific laboratory abnormalities associated with his Legionnaire’s disease, e.g., hypophosphatemia, elevated ferritin, elevated creatinine phosphokinase CPK. He remained afebrile after doxycycline therapy, and levofloxacin was used to complete therapy of Legionnaire’s disease. During the remainder of his NICU stay, serial chest films were done to detect potential *B.* *cepacia* pneumonia, but chest films remained clear without infiltrates.

Repeat cultures of his respiratory secretions revealed heavy growth of *B. contaminans* susceptible to levofloxacin. Serial Gram stains and cultures of his respiratory secretions were obtained to monitor possible antibiotic resistance and to assess the effectiveness of levofloxacin on decreasing the intensity and duration of *B.* *contaminans* carriage.

These concerns had important Infection Control and Antibiotic Stewardship Program (ASP) implications. Liquid docusate (Colace), from the two bottles (bottles #1 and #2) given to the patient and the third bottle (bottle #3) intended for the patient (unused/closed), but not dispensed, were cultured. Bottle #1 (opened/unused) grew abundant *B. contaminans*, and bottle #2 (the other used/opened bottle) grew (heavy growth) *C. pelliculosa*. Bottle #3 (unopened/used) also grew *C.* *pelliculosa*. *C. pelluosa* cultured from liquid docusate (Colace) bottles (bottle #2: open/used; bottle #3: closed/unused) was never cultured from the patient’s respiratory secretions. *C. pelliculosa* was identified using API20C AUX bio Merieux Vitek, Inc., Hazelwood, MO, USA.

NICU staff was educated by Infection Control on the importance of strict contact precautions to prevent *B.* *contaminans* spread to other patients in the ICU and the hospital. The prolonged persistence of increasingly multidrug-resistant (MDR) *B. contaminans* in the respiratory secretions of the index case was concerning. From an infectious disease standpoint, colonization is not usually treated. From an ASP perspective, treatment of colonization usually fails, and in the process, the organism not only persists but may become resistant or more MDR.

Because of the potential for nosocomial spread of MDR GNB in the NICU (and hospital), it was decided to try to eliminate *B. contaminans* colonization from the patient’s respiratory secretions with trimethprim-sulfamethoxazole (TMP-SMX). TMP-SMX was selected because it was the one of the few antibiotics remaining that was effective against this strain of *B.* *contaminans* and because of its ability to penetrate into respiratory secretions well. Although his strain of *B. contaminans* remained susceptible to TMP-SMX, TMP-SMX (10 mg/kg/day) failed to eliminate *B. contaminans* colonization from his respiratory secretions. However, the concentration (numbers) of *B. contaminans* in his respiratory secretions decreased over time, minimizing the potential for spread to other NICU patients.

Since the index case, ongoing Infection Control and microbiology laboratory surveillance has revealed no further *B.* *contaminans* isolates in our 600-bed university-affiliated teaching hospital. The patient was eventually transferred to a chronic care facility for rehabilitation of his neurologic deficits.

First identified as a separate BCC species in 2009 by DNA testing, *B. contaminans* has been isolated from soil, water, and contaminated medications [12,13,14,15,16,17,18]. While BCC species have been described in a variety of nosocomial outbreaks, *B. contaminans* has been reported in isolated cases, but only rarely in hospital outbreaks. BCC species are well-known opportunistic pathogens in cystic fibrosis (CF) patients [14,15,16,17]. In the past, the most frequently isolated BCC species have been *B. mulitvorans* and *B. cenocepacia*. However, over the past decade, *B. contaminans* has emerged in the most frequent BCC species. BCC species differ in their clinical manifestations and preferred hosts. *B. multivorans* and *B. cenocepacia* have been the most frequent species colonizing/infecting CF patients [13,14]. BCC colonization alone decreases pulmonary function in CF patients. In contrast, *B. contaminans* has emerged as a bona fide pathogen causing bacteremia or necrotizing pneumonia in critically ill non-cystic fibrosis patients [14,19,20]. *B. contaminans* may gain access to respiratory secretions or the bloodstream in a variety of ways, e.g., *B. contaminans*–contaminated fluids. Unlike *B. cenocepacia* and *B.* *multivorans,* *B.* *contaminans* is relatively more susceptible to antibiotics, but may become MDR. During treatment, an important characteristic of BCC species is their ability to colonize/persist in fluids including respiratory secretions. In non-CF patients, *B. contaminans* isolated from blood or respiratory secretions should alert Infectious Control and clinicians to the possibility of a common source of BCC-contaminated fluids, particularly in ventilated or critically ill patients. BCC-contaminated fluids include mouthwash, chlorhexidine, saline solutions, nasal sprays, inhaled medications, heparin and intravenous solutions. BCC also has the potential to present as a pseudoinfection, i.e., pseudobacteremia [18,21].

In hospitalized patients, colonization of body fluids with MDR GNB is ordinarily contained by Infectious Control contact precautions and usually not “treated” with antibiotics. Since BCC species are difficult to eradicate from respiratory secretions, prolonged antibiotic therapy often not only fails to eliminate the organism from secretions, but the strain often becomes more resistant over time.

*B. contaminans*, like other BCC species, is a fastidious GNB. In the clinical microbiology laboratory, two days are required for the identification of BCC. DNA testing is required for *B. contaminans* speciation. Limiting the spread of MDR GNB, e.g., *B. contaminans* in respiratory secretions of critically ill patients, is an ongoing Infectious Control challenge. Although the usual manifestation of *B. contaminans* is colonization rather than infection, there have been reports of bacteremia, sepsis, and necrotizing pneumonia due to this organism in non-CF critically ill or immunosuppressed patients.

From an infection disease perspective, effective eradication of *B. contaminans* depends on selecting an antibiotic with a high degree of inherent activity against the organism and the antibiotic should have a “low resistance” potential [22,23]. Since prolonged therapy is usually required to eliminate *B. contaminans* carriage from secretions, resistance potential is an important therapeutic consideration. The antibiotic selected should also possess the requisite physiochemical and pharmacokinetic properties that permit penetration into respiratory secretions in therapeutic concentrations, i.e., antibiotics with a high volume of distribution (*V_d_*) > 0.7 L/kg. Taking these factors into account, the preferred antibiotics for *B. contaminans* eradication in respiratory secretions include levofloxacin, minocycline, and TMP-SMX. Other antibiotics that initially may be reported as susceptible for *B. contaminans*, e.g., ceftazidime, have a “high resistance potential” and are prone to develop resistance. In addition, ceftazidime’s low *V_d_* < 0.7 L/kg predicts its poor penetration into respiratory secretions. Other things being equal, subtherapeutic tissue concentrations predispose the development of MDR GNB [22,23]. In our case, following doxycycline, levofloxacin was used to complete the course of therapy for Legionnaire’s disease. After resolution of his Legionnaire’s disease, levofloxacin was continued with the intent of eliminating *B. contaminans* from the patient’s respiratory secretions. However, during levofloxacin therapy, *B. contaminans* persisted in his respiratory secretions and became more resistant. TMP-SMX was given in an attempt to decrease/eliminate *B. contaminans* from his respiratory secretions in order to minimize the potential for nosocomial spread. Effective Infection Control contact precautions to contain the MDR GNB in the NICU were repeatedly stressed to the staff. During TMP-SMX therapy, there was neither further loss of susceptibility nor increased resistance. TMP-SMX decreased the concentration of *B. contaminans* in his respiratory secretions and he never developed *B. contaminans* pneumonia or bacteremia. However, TMP-SMX was unable to eradicate the carriage of *B. contaminans* from his respiratory secretions (Table 1).

Another intriguing microbiologic aspect of this case was the significance of *C. pelliculosa* isolated from two bottles (bottles #2 and #3) of liquid docusate (Colace). Not only is *C. pelliculosa* an extremely rare *Candida* sp., it was isolated from two of three liquid docusate (Colace) bottles, some of which was administered to the patient (from bottle #2). *C. pelliculosa* was also isolated from bottle #3 (unused/unopened) of liquid docusate (Colace). It is noteworthy that some *B. contaminans* strains, e.g., MS 14, produce a potent anti-fungal glycopeptide, occidiofungin. Interestingly, *C. pelliculosa* did not grow from bottle #1 containing *B. contaminans.* However, bottles #2 and #3 both grew *C. pelliculosa,* but *B. contaminans* did not grow from either bottle #2 or bottle #3.

*C. pelliculosa* is a yeast and is the asexually reproducing anamorph of *Wickerhamomyces anomalus* (formerly *Hansenula anomala* and *Pichia amomala*) found in soil, grain, water, sewage, and especially fermenting fruit. *C. pelliculosa* is important in flavor enhancement, food processing, dairy fermentation and waste water treatment. *C. pelliculosa* has been found to be associated with the malaria vector *Anopheles stephensi* in Asia [19,20]. In recent years, *C. pelliculosa* has been used as a biocontrol agent due to its ability to produce potent mycotoxins, e.g., occidiofungin [24,25]. *C. pelliculosa* has been reported to cause keratitis in corneal transplants, arthritis, meningitis (in HIV), and acute pancreatitis in bone marrow and solid organ transplants, as well as nosocomial infections in neonatal and pediatric ICUs. Risk factors associated with *C. pelliculosa* infection include central venous catheters (CVCs) and total parenteral nutrition (TPN). In sickle cell patients, *C. pelliculosa* may cause urinary tract infections or fungemia in critically ill or immunocompromised patients [19,20].

It is interesting to speculate that some lots of liquid docusate (Colace) contaminated with *B. contaminans* may have also been contaminated with *C. pelliculosa. C. pelliculosa*–contaminated liquid docusate (Colace) bottles may have inhibited the growth of *B. contaminans.* We suggest that hospitals with outbreaks of *B. cepacia*/*B. contaminans* involving contaminated lots of liquid docusate (Colace) should also be alert to the potential of *C. pelliculosa* as well as *B. contaminans* intrinsic contamination. Bottles contaminated with *C. pelliculosa* may have also contained *B. contaminans* which may not have grown if the strain of *B. contaminans* involved produced occidiofungin and inhibited the growth of *B. contaminans*.

Firstly, the CDC was critical in alerting us to the ongoing multistate *B. cepacia* (*B*. *contaminans* in this case) outbreaks. Secondly, *B. contaminans* is an “unusual” nosocomial isolate that may cause colonization or infection in non-CF, particularly critically ill or ventilated patients. Thirdly, as a fastidious aerobic GNB, *B. contaminans* requires two days for microbiologic identification as BCC. Fourth, while not highly virulent or invasive, *B. contaminans* may persist in aqueous environments, e.g., the patient’s secretions or body fluids as well as a variety of aqueous solutions, such as liquid docusate (Colace) in this instance. Fifth, patient exposure to *C. pelliculosa* from bottle #2 of the liquid docusate (Colace) did not result in the colonization of his respiratory secretions. Sixth, GNB colonization is difficult to eliminate even with antibiotics highly active against the organism, even when the antibiotic penetrates well into secretions, e.g., levofloxacin, TMP-SMX. Seventh, strict Infection Control contact precautions remain the cornerstone of containing MDR GNB, e.g., *B. contaminans* colonization and spread in the ICU and the hospital. Lastly, following the CDC alert, stopping further patient exposure was rapidly achieved by conducting epidemiologic and microbiologic investigations, and instituting strict Infection Control containment measures. These patient-protective measures were possible due to the rapid response of effective leadership and the coordination of several key individuals and hospital departments, e.g., the hospital epidemiologist, the Infection Control department, the NICU nursing staff, the medical microbiology laboratory, the pharmacy department, and the Infectious Disease Division.

As in all such cases, some unanswered questions remain. In our experience, the index patient was exposed to two contaminated liquid docusate (Colace) bottles, i.e., one that grew *B. contaminans* (bottle #1) and the other that grew *C.* *pelliculosa* (bottle #2), but only *B. contaminans* colonized the patient’s respiratory secretions. Could potential additional exposure to *C. pelliculosa*–containing liquid docusate (Colace) (bottle #3: unopened/unused) have resulted in *C. pelliculosa* colonization? As the multistate outbreak continues, Infection Control, medical microbiology and infectious disease personnel should also be alerted to the growth-suppression potential of *C. pelliculosa* in bottles of liquid docusate (Colace) containing *B. contaminans*.

## Figures and Tables

**Table 1:** Serial Gram stains and cultures of respiratory secretions.

Hospital Day:	HD #13 ^†^	HD #17		HD #20		HD #23		HD #26		HD #29		HD #31	
**Gram Stain: (WBCs)**	WBCS: Few Epithelial cells: many	WBCS: Some Epithelial cells: None		WBCS: Few Epithelial cells: Few		WBCS : Few Epithelial cells: Few		WBCS: Few Epithelial cells: Few		WBCS: Few Epithelial cells: Few		WBCS: Few Epithelial cells: Few	
**Gram Stain: (Organisms)**	Gram-negative bacilli: Few	No Organisms Seen		Gram-positive cocci in pairs: Few Gram-negative bacilli: Many		Gram-negative bacilli: Few		Gram-positive cocci in pairs : Few in chains: Few in clusters : Few Gram-negative bacilli: Many		Gram-positive cocci in clusters: Few		Gram-negative bacilli: Very Few	
**Culture Results**	Normal throat flora	**Burkholderia contaminans** (++) Candida albicans: Few		**Burkholderia contaminans** (+++)		**Burkholderia contaminans** (+++)		**Burkholderia contaminans** (+++++)		**Burkholderia contaminans** (++)		**Burkholderia contaminans** (+) Candida albicans: Few	
**Antibiotic**	**Doxycycline**	**Levofloxacin**		**Levofloxacin**		**Levofloxacin**		**Levofloxacin**		**TMP-SMX**		**TMP-SMX**	
**Antibiotic Susceptibility**	Not Applicable	Ampicillin	>16 R	Ampicillin	>16 R	Ampicillin	>16 R	Ampicillin	>16 R	Ampicillin	>16 R	Ampicillin	>16 R
		Amikacin	>32 R	Amikacin	>32 R	Amikacin	>32 R	Amikacin	>32 R	Amikacin	>32 R	Amikacin	>32 R
		**Ceftazidime**	**8 S**	**Ceftazidime**	**>16 R**	**Ceftazidime**	**8 S**	**Ceftazidime**	**8 S**	**Ceftazidime**	**8 S**	**Ceftazidime**	**8 S**
		**Ciprofloxacin**	**<=1 S**	**Ciprofloxacin**	**<=1 S**	**Ciprofloxacin**	**>4 R**	**Ciprofloxacin**	**>4 R**	**Ciprofloxacin**	**>4 R**	**Ciprofloxacin**	>4 R
		Cefepime	>16 R	Cefepime	>16 R	Cefepime	>16 R	Cefepime	>16 R	Cefepime	>16 R	Cefepime	>16 R
		Ceftriaxone	>32 R	Ceftriaxone	>32 R	Ceftriaxone	>32 R	Ceftriaxone	>32 R	Ceftriaxone	>32 R	Ceftriaxone	>32 R
		**Doxycycline**	**>8 R**	**Doxycycline**	**>8 R**	Doxycycline *	**>8 R**	Doxycycline *	**>8 R**	Doxycycline *	**>8 R**	Doxycycline *	**>8 R**
		Gentamicin	>8 R	Gentamicin	>8 R	Gentamicin	>8 R	Gentamicin	>8 R	Gentamicin	>8 R	Gentamicin	>8 R
		**Levofloxacin**	**<=2 S**	**Levofloxacin**	**=2 S**	**Levofloxacin**	**>32 R**	**Levofloxacin**	**>32 R**	**Levofloxacin**	**>32 R**	**Levofloxacin**	**>32 R**
		**Meropenem**	**<=1 S**	**Meropenem**	2 S	**Meropenem**	2 S	**Meropenem**	<=1 S	**Meropenem**	2 S	Meropenem	<=1 S
		Pip/Tazo	<=16 S	Pip/Tazo	64 I	Pip/Tazo	<=16 S	Pip/Tazo	<=16 S	Pip/Tazo	<=16 S	Pip/Tazo	<=16 S
		**TMP-SMX**	**<=2/38 S**	**TMP-SMX**	**<=2/38 S**	**TMP-SMX**	**<=2/38 S**	**TMP-SMX**	**<=2/38 S**	**TMP-SMX**	**<=2/38 S**	**TMP-SMX**	**<=2/38 S**

pip/tazo = piperacillin/tazobactam; * minocycline MIC (Etest) = 2 µg/mL; ^†^ Patient given liquid docusate (Colace) on HD #7 and HD #8.
